# Congestion, but not low cardiac output, is independently associated with acute kidney injury following contrast agent exposure

**DOI:** 10.3389/fcvm.2026.1797135

**Published:** 2026-06-15

**Authors:** Jean Marc Haurand, Elric Zweck, Patrick Horn, Christian Jung, Malte Kelm, Frank Ruschitzka, Ralf Westenfeld, Amin Polzin

**Affiliations:** 1 Department of Cardiology, Pulmonology, and Vascular Medicine, Medical Faculty, Heinrich Heine University, Düsseldorf, Germany; 2 Cardiovascular Research Institute Düsseldorf, Medical Faculty, Heinrich Heine University, Düsseldorf, Germany; 3 Department of Cardiology, University Hospital Zurich, Zurich, Switzerland

**Keywords:** acute kidney injury, backward failure, forward failure, low output, MEHRAN score, venous congestion

## Abstract

**Aim:**

Contrast-associated acute kidney injury (CA-AKI) is frequently associated with adverse outcomes. Renal perfusion gradient is central to glomerular filtration. While renal vasoconstriction can partially compensate low-output-associated impairment of the renal perfusion gradient, venous congestion cannot be compensated. We hypothesized that congestion is a stronger predictor of CA-AKI than low output in cardiovascular diseases.

**Methods and results:**

An all-comer single-center cohort of 3,126 cases undergoing coronary angiography with simultaneous right heart catheterization was analyzed. Patients were divided into four categories: congested [right atrial pressure (RAP) >10 mmHg and cardiac index (CI) >=2.2 L/min/m^2^], low output (RAP <= 10 mmHg and CI: <2.2 L/min/m^2^), combined (RAP > 10 mmHg and CI <2.2 L/min/m^2^), and control (CI >= 2.2 L/min/m^2^ and RAP <= 10 mmHg). The average age of patients was 74 years. CA-AKI occurred in 19% of patients overall. The incidence was highest among patients with congestion, who showed a significantly greater risk compared to controls (24% vs. 15%; Hazard Ratio (HR): 1.49, *p* = 0.001), whereas low-output patients showed only a trend towards increased risk (20% vs. 15%; HR: 1.22, *p* = 0.059). In multivariate analysis including the MEHRAN Score, only congestion but not low output was independently associated with AKI (HR for 5 mmHg increase in RAP: 1.07, *p* = 0.034; HR for CI: 1.00, *p* = 0.936).

**Conclusions:**

Venous congestion is independently associated with CA-AKI and is a stronger predictor of CA-AKI than low cardiac output.

## 1 Introduction

Chronic kidney disease (CKD) is a frequent comorbidity in patients with cardiovascular disease (CVD) (1–3). Impaired renal function is linked to the progression of coronary artery disease (CAD), heart failure, and arrhythmias, resulting in higher morbidity and mortality in patients with both CVD and CKD (1, 4–7). Beyond age and CKD, acute kidney injury (AKI) in CVD is associated with an additional increase in mortality (8, 9). Administration of contrast agents is associated with AKI (10, 11). In this context, the MEHRAN score is the gold standard for risk stratification to estimate the probability of contrast-associated AKI (CA-AKI) (12). Several interactions between CKD and CVD lead to mutual deterioration (13). Considering the renal perfusion gradient as the backbone of glomerular filtration, both low cardiac output and venous congestion are associated with AKI incidence and decreased estimated glomerular filtration rate (eGFR) in patients with CVD (6, 14–16). The autoregulatory effects of the kidney can compensate for the hemodynamic states of chronically impaired cardiac output, whereas venous congestion cannot be compensated (17, 18). Therefore, in this study, we investigated the association between venous congestion and CA-AKI in both the presence and absence of low cardiac output. We hypothesized that (i) both venous congestion and cardiac output are associated with baseline eGFR and occurrence of CA-AKI in patients undergoing right heart catheterization (RHC) and (ii) venous congestion is a superior predictor of CA-AKI than low cardiac output.

## 2 Methods

We conducted an analysis of the Düsseldorf quality assurance database at the University Hospital of Düsseldorf, Germany. In this retrospective, single-center cohort study, all patients undergoing RHC between 2018 and 2023 were included. Exclusion criteria were chronic need for hemodialysis prior to admission, missing hemodynamic data for right atrial pressure (RAP) and cardiac index (CI), cases without recorded baseline eGFR, and known contrast agent exposure within 30 days prior to RHC.

This study was conducted in accordance with the Declaration of Helsinki and was approved by the University of Düsseldorf Committee on Human Research (study number 2018-48). All parameters used were collected as part of routine clinical practice in a registry, and informed consent was obtained.

**Figure 1:**
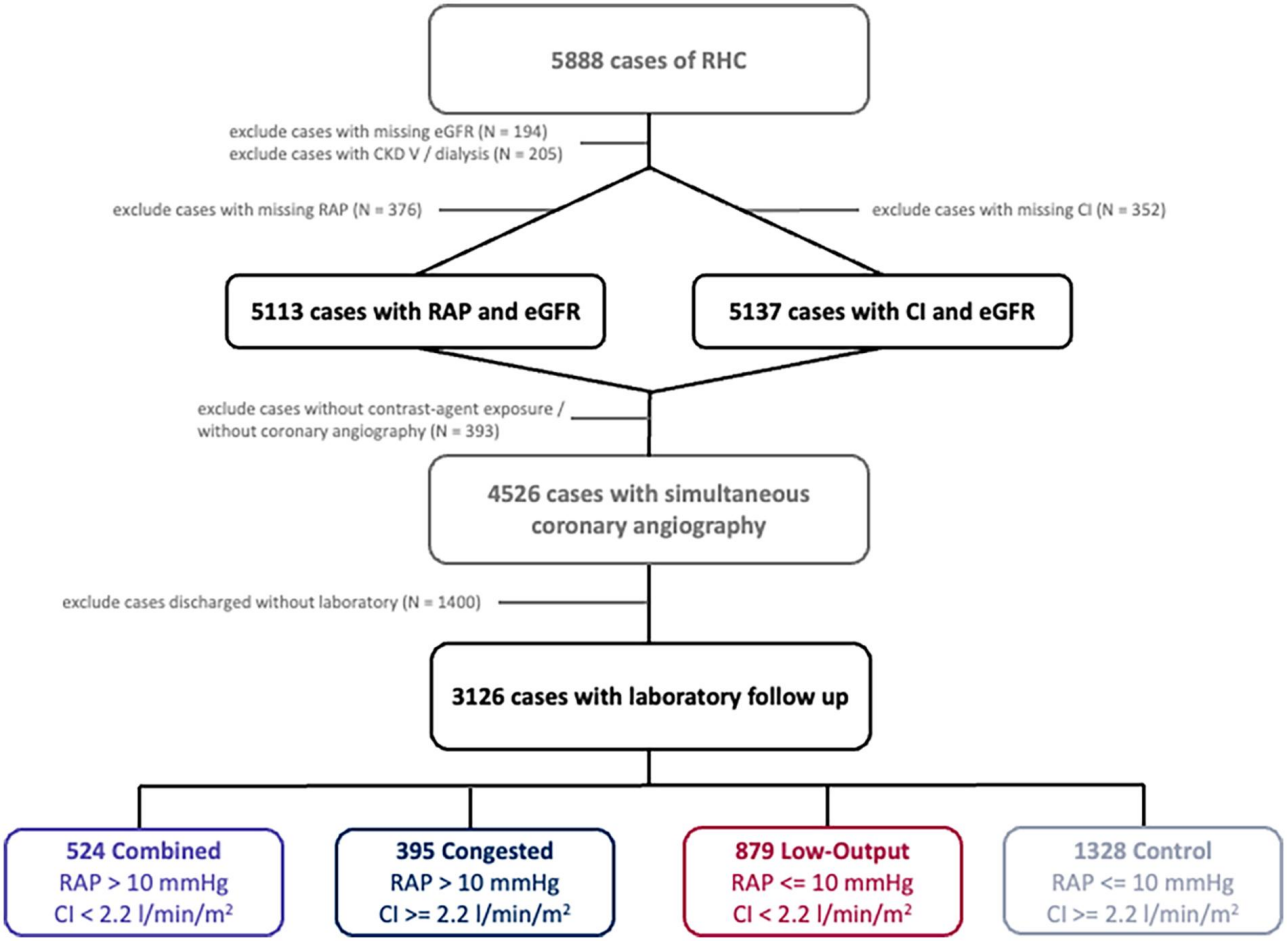
Study flowchart. Study flowchart showing inclusion and exclusion criteria of cases and subgroups. CI, cardiac index; CKD, chronic kidney disease; eGFR, estimated glomerular filtration rate; KDIGO, kidney disease improving global outcomes; RAP, right atrial pressure; RHC, right heart catheterization.

Detailed exclusion criteria and patient grouping are shown in the study flowchart (Figure 1).

The primary exposure in this analysis was the hemodynamic category, defined by RAP measured by RHC and CI, defined by cardiac output (calculated with the Fick principle) per body surface area (calculated using the DuBois formula): (i) *Congestion*, with RAP > 10 mmHg and CI ≥ 2.2 L/min/m^2^; (ii) *Low output*, with RAP ≤ 10 mmHg and CI < 2.2 L/min/m^2^; (iii) *Combined*, with RAP > 10 mmHg and CI < 2.2 L/min/m^2^; and (iv) *Control*, with CI ≥ 2.2 L/min/m^2^ and RAP <= 10 mmHg.

For analysis of the primary outcome CA-AKI, only cases with simultaneous contrast agent exposure during coronary angiography were included. Patients without repeated creatinine measurements or discharged without laboratory follow-up were excluded. AKI was defined according to KDIGO criteria (19). The endpoint CA-AKI was defined as the time of the first laboratory creatinine measurement fulfilling the modified KDIGO criteria for AKI (increase in creatinine of 0.3 or greater within 48 h or an increase by factor 1.5 within 7 days compared with creatinine at baseline) (19). Creatinine and eGFR at baseline were defined by the lowest documented creatinine value up to 90 days prior to coronary angiography with RHC. In a sensitivity analysis, we defined baseline only by the lowest documented creatinine value up to 7 days prior to RHC. Baseline eGFR was calculated using the CKD-EPI formula by Levey et al. (20).

**Figure 2:**
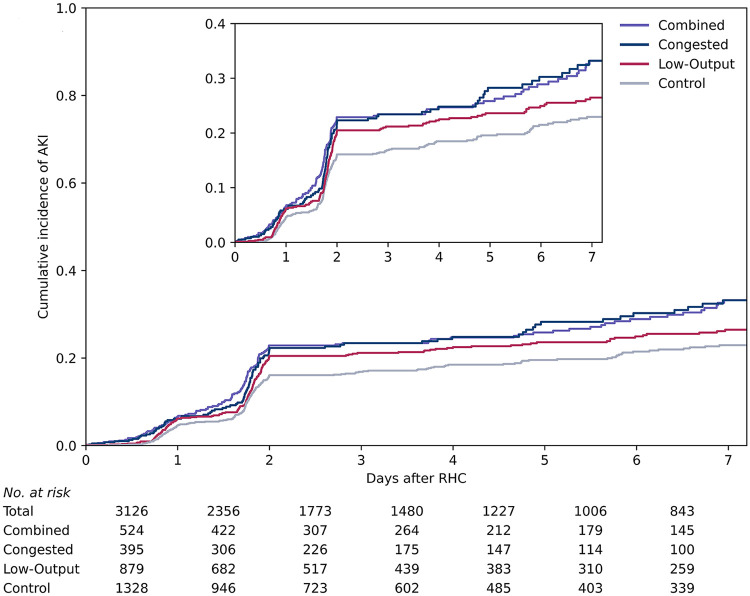
Incidence of CA-AKI. Kaplan–Meier survival curves with cumulative incidence of contrast-associated acute kidney injury (CA-AKI) within 7 days following coronary angiography with right heart catheterization in four different hemodynamic categories. Relative frequency of CA-AKI in the combined subgroup (*n* at risk at baseline 524) is 24%, in congested (*n* at risk at baseline 395) is 24%, in low output (*n* at risk at baseline 879) is 20%, and in control (*n* at risk at baseline 1,328) is 15%. The number at risk for baseline day 0 to day 6 for the cohort (*n* = 3,126) is listed in the table.

**Figure 3:**
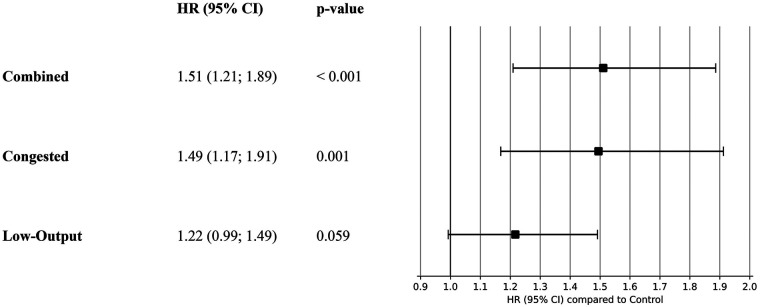
CA-AKI in hemodynamic categories. Univariate cox regression analysis for time-dependent outcome of CA-AKI in full cohort (*n* = 3,126) for categories combined, congested, and low output versus reference control. Combined vs. control HR: 1.51 (95% CI: 1.21–1.89, *p* < 0.001), congested vs. control HR: 1.49 (95% CI: 1.17–1.91, *p* = 0.001), and low output vs. control HR: 1.22 (95% CI: 0.99–1.49, *p* = 0.059).

**Figure 4:**
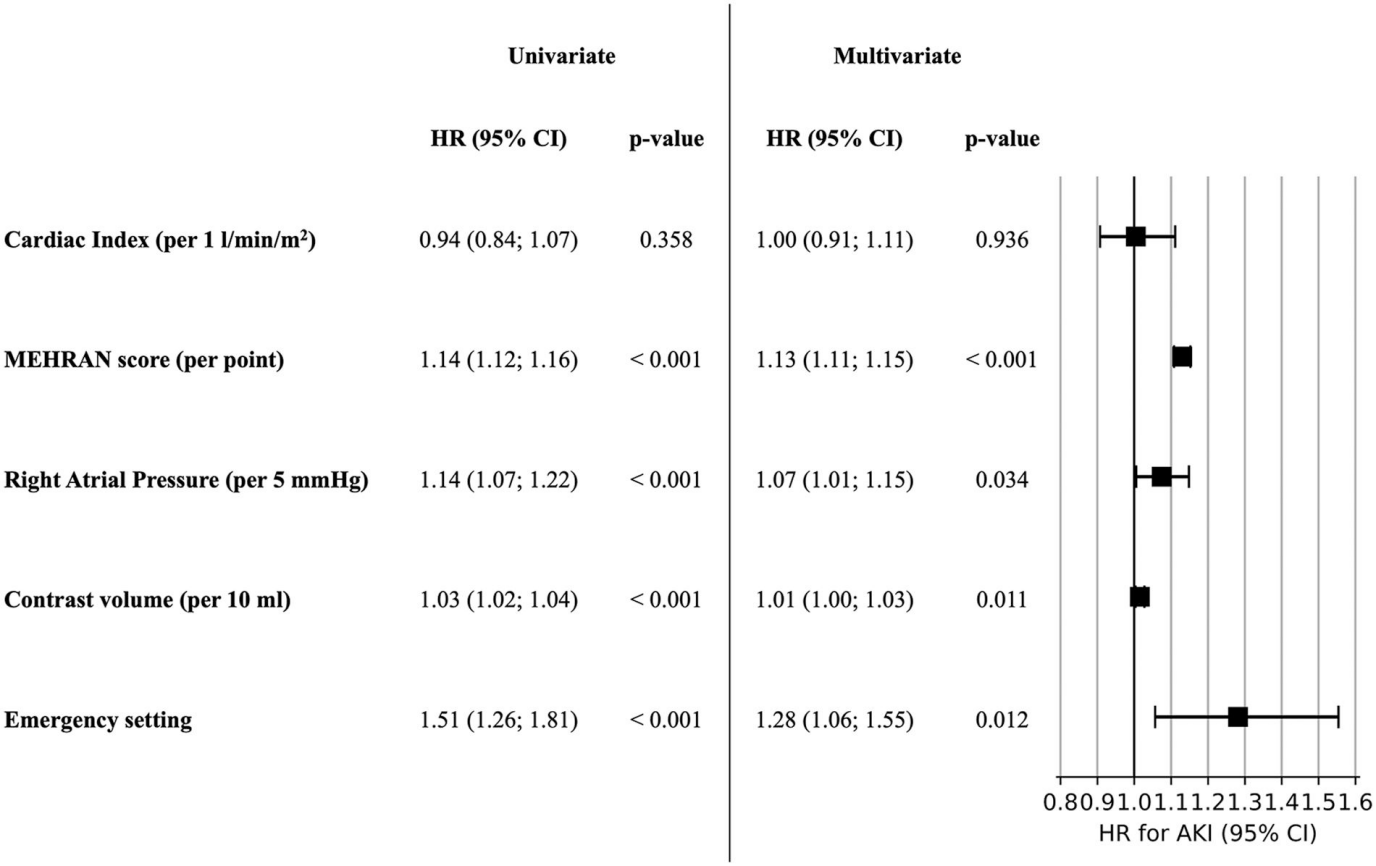
Association of RAP and CI with CA-AKI. Univariate and multivariate cox regression analyses for time-dependent outcome of CA-AKI in full cohort (*n* = 3,126), with factors cardiac index, MEHRAN score (including age, systemic blood pressure, prevalence of left ventricular dysfunction, contrast media volume, diabetes mellitus, anemia, and state of chronic kidney disease), RAP (per 5 mmHg), contrast agent volume (per 10 mL), and emergency setting (urgent procedure due to acute coronary syndrome). Univariate HR for increase of cardiac index per 1 L/min/m^2^ 0.94 (95% CI: 0.84–1.07, *p* = 0.358), HR for increase of MEHRAN score per point 1.14 (95% CI: 1.12–1.16, *p* < 0.001), HR for increase of RAP per 5 mmHg 1.14 (95% CI: 1.07–1.22, *p* < 0.001), HR for increase of contrast volume per 10 mL 1.03 (95% CI: 1.02–1.04, *p* < 0.001), and HR for emergency setting 1.51 (95% CI: 1.26–1.81, *p* < 0.001). Multivariate model with all-factor HR for increase of RAP per 5 mmHg 1.07 (95% CI: 1.01–1.15, *p* = 0.034), adjusted for cardiac index (HR: 1.00, 95% CI: 0.91–1.11, *p* = 0.936), MEHRAN score (HR per point 1.13 (95% CI: 1.11–1.15, *p* < 0.001), contrast volume (HR per 10 mL 1.01, 95% CI: 1.00–1.03, *p* = 0.011), and emergency setting (HR: 1.28, 95% CI: 1.06–1.55, *p* = 0.012).

**Figure 5:**
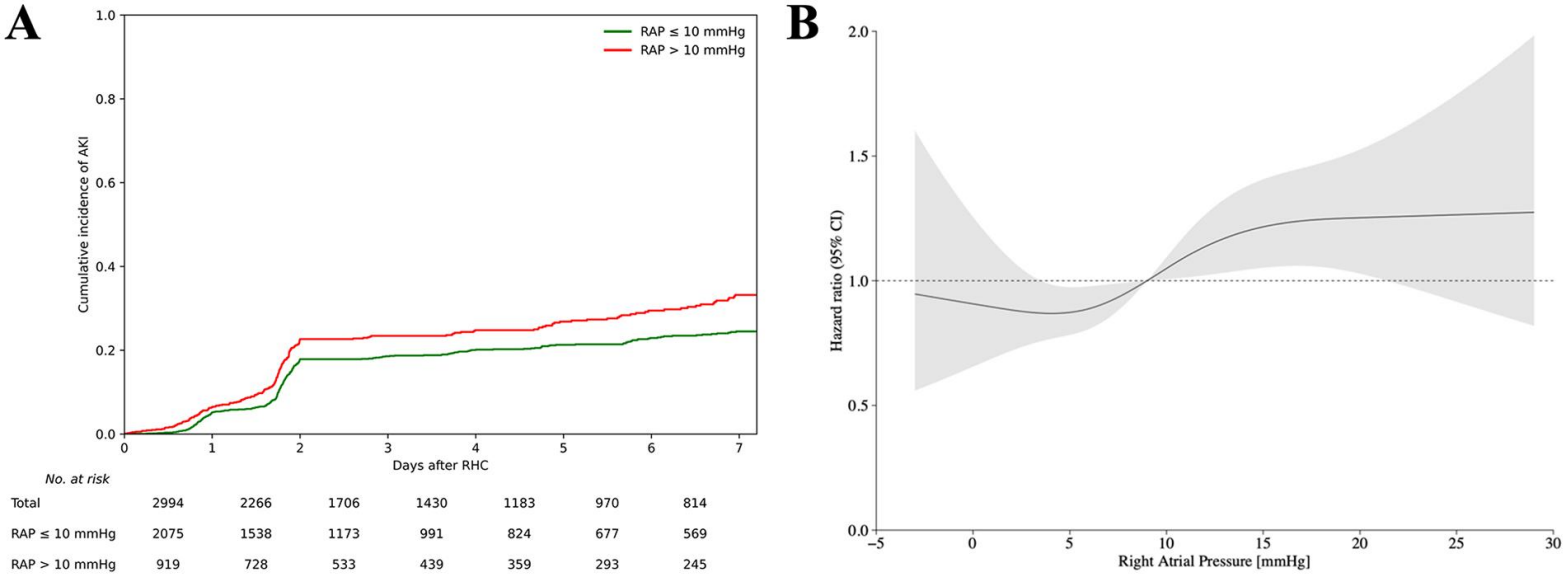
Hazard ratio of AKI stratified by RAP. **(A)** Kaplan–Meier survival curves with cumulative incidence of CA-AKI within 7 days following coronary angiography with right heart catheterization in cases with right atrial pressure (RAP) > 10 mmHg and ≤10 mmHg. **(B)** Restricted cubic spline regression for hazard ratio of acute kidney injury (AKI) across the range of RAP with 95% confidence interval (light gray), using 4 knots between 0.05 and 0.95 quantiles, with reference at cohort mean RAP.

To visualize the time-dependent occurrence of AKI, Kaplan–Meier survival curves depicting cumulative occurrence of CA-AKI per hemodynamic category were generated (Figure 2). Hemodynamic categories were compared with the reference control group using univariate Cox regression (Figure 3). For prediction of time-dependent occurrence of CA-AKI, univariate and multivariate Cox regression models were used (Figure 4). The MEHRAN score was assessed, incorporating several risk factors for occurrence of CA-AKI (systolic blood pressure/use of inotropes or mechanic circulatory support, heart failure with NYHA III or IV or history of pulmonary edema, age, anemia, diabetes, contrast agent volume, and eGFR at baseline) (12). Urgent procedures performed in the context of acute coronary syndrome were classified as “emergency setting.” To assess the impact of congestion, only Kaplan–Meier curves stratified by RAP (>10 and ≤10 mmHg) were drawn (Figure 5A). Partial hazard ratios among varying values of RAP, adjusted for MEHRAN score and CI, were calculated using multivariate Cox regression with restricted cubic splines with four knots (Figure 5B).

In addition, we assessed the relationship between the hemodynamic parameters RAP and CI and eGFR at baseline (Supplementary Figure S1). Age- and gender-adjusted cubic polynomial regression models were fitted using ordinary least squares. The optimal polynomial degree was determined by fitting models with second-, third-, and fourth-order terms, selecting the best fit based on F-statistics and likelihood criteria. To illustrate the incidence of CA-AKI as a function of contrast agent volume, Kaplan–Meier curves were plotted, stratified by contrast agent volume.

Two-sided *p* values were reported, with a *p* < 0.05 considered statistically significant.

Statistical analyses were performed using the scikit-learn package (version 1.2.2), SciPy package (version 1.10.1), and lifelines package (version 0.27.4) in python (version 3.10.9), as well as R version 4.3.0 (R Foundation for Statistical Computing, Vienna, Austria). Categorical variables are reported as absolute values and percentages, whereas continuous data are expressed as means with standard deviation.

## 3 Results

### 3.1 Definition and characteristics of cohort

Out of a total of 5,888 cases of RHC between 2018 and 2023, 3,126 cases had simultaneous coronary angiography with contrast agent exposure and laboratory follow-up (Figure 1). The mean age was 74 years, and 41% of patients were women. The mean body mass index was 27 kg/m^2^. Most patients had CKD stage G2 (42%), followed by stage G3a (22%), stage G3b (17%), G1 (12%), and stage G4 (8%). The main indications were evaluation of coronary artery disease in 94% of the cases, often overlapping with varying indications such as evaluation of valvular heart diseases (64% of cases), with aortic stenosis in 42% being the most common. Other indications included new onset of heart failure or evaluation of chronic heart failure, with preserved or reduced ejection fraction in 44% of the cases.

Detailed demographic, comorbidity, and diagnostic data, as well as indications for coronary angiography with RHC, and relevant laboratory values are presented in Table 1. Data from the full screening cohort undergoing RHC are provided in Supplementary Table S2.

**Table 1:** Characteristics of study population.

Demographics		Missing values, *n* (%)
Age (years), mean (SD)	74.4 (12.3)	0
BMI (kg/m^2^), mean (SD)	27.1 (5.3)	0
Gender female, *n* (%)	1,273 (40.7)	0
Diabetes mellitus, *n* (%)	409 (13.1)	0
Hypertension, *n* (%)	1,209 (38.7)	0
Hyperlipidemia, *n* (%)	1,009 (32.3)	0
Anemia, *n* (%)	1,319 (42.2)	0
CKD		0
G1, *n* (%)	366 (11.7)	
G2, *n* (%)	1,305 (41.7)	
G3a, *n* (%)	695 (22.2)	
G3b, *n* (%)	522 (16.7)	
G4, *n* (%)	238 (7.6)	
Diagnoses and indications for coronary angiography and RHC		Missing values, *n* (%)
Coronary angiography, *n* (%)	2,950 (94.4)	0
CAD, *n* (%)	2,238 (73.0)	
Acute coronary syndrome, *n* (%)	591 (18.9)	
Out hospital cardiac arrest, *n* (%)	52 (1.7)	
Valvular heart disease, *n* (%)	2,002 (64.0)	0
Aortic stenosis, *n* (%)	1,308 (41.8)	
Aortic regurgitation, *n* (%)	68 (2.2)	
Mitral regurgitation, *n* (%)	745 (23.8)	
Tricuspid regurgitation, *n* (%)	155 (5.0)	
Pulmonary stenosis, *n* (%)	7 (0.2)	
Heart failure, *n* (%)	1,364 (43.6)	0
HFpEF, *n* (%)	90 (2.9)	
HFmrEF, *n* (%)	450 (14.4)	
HFrEF, *n* (%)	763 (24.4)	
DCM, *n* (%)	141 (4.5)	
H(O)CM, *n* (%)	39 (1.2)	
Heart transplant, *n* (%)	173 (5.5)	
Biopsy, *n* (%)	231 (7.4)	
Peri-/myocarditis, *n* (%)	44 (1.4)	
Cardiogenic shock, *n* (%)	20 (0.6)	
Others, *n* (%)	152 (4.9)	0
Shunt diagnostic, *n* (%)	20 (0.6)	
PAH, *n* (%)	39 (1.2)	
Pericardial effusion, *n* (%)	60 (1.9)	
Laboratory values and scores		Missing values, *n* (%)
Creatinine (mg/dL), mean (SD)	1.2 (0.5)	0
eGFR (mL/min), mean (SD)	61.4 (22.4)	0
Days between baseline laboratory measurement and RHC, mean (SD)	3.3 (10.8)	0
Cystatin C (mg/L), mean (SD)	1.7 (0.8)	2,061 (66)
Urea (mg/dL), mean (SD)	49.8 (26.8)	0
NT-pro-BNP (pg/mL), mean (SD)	3,695.2 (6,086.7)	550 (18)
HbA1c (%), mean (SD)	6.1 (1.1)	1,190 (38)
Hemoglobin (g/dL), mean (SD)	12.7 (2.1)	0
Hematocrit (%), mean (SD)	39.2 (5.9)	0
MEHRAN score, mean (SD)	6.7 (4.0)	0
Contrast volume (mL), mean (SD)	89.9 (60.8)	0
Contrast volume/eGFR, mean (SD)	1.7 (1.6)	0

BMI, body mass index; CAD, coronary artery disease; CKD, chronic kidney disease; DCM, dilated cardiomyopathy; HFmreF, heart failure with midrange ejection fraction; HFpEF, heart failure with preserved ejection fraction; HFrEF, heart failure with reduced ejection fraction; H(O)CM, hypertrophic (obstructive) cardiomyopathy; PAH, pulmonary arterial hypertension; eGFR, estimated glomerular filtration rate.

Characteristics of cohort for primary exposure (*N* = 3,126), including demographics, diagnoses/indications for right heart catheterization, and selected laboratory values at baseline. Values are mean with standard deviation or number with relative value.

The mean RAP was 8.2 mmHg, and the mean CI was 2.4 L/min/m^2^. Hemodynamic parameters from RHC and additional echocardiographic values are shown in Supplementary Table S1, with full cohort data of RHC cases in Supplementary Table S3. Correlations between different hemodynamic, echocardiographic, and laboratory values are illustrated in Supplementary Figure S2.

### 3.2 Occurrence of CA-AKI in different hemodynamic categories

We observed the occurrence of CA-AKI across four hemodynamic categories. The overall frequency of CA-AKI was 19%. The relative frequency of CA-AKI was 24% in both the combined subgroup (*N* = 524) and the congested subgroup (*N* = 395).. In the low-output subgroup (*N* = 879), CA-AKI occurred in 20% of the cases. The control subgroup (*N* = 1,328) had the lowest rate of CA-AKI with 15%. The cumulative incidence of CA-AKI is depicted in Figure 3. The rate of CA-AKI was higher in the congested (HR: 1.49; 95% CI: 1.17–1.91; *p* = 0.001) and combined (HR: 1.51; 95% CI: 1.21–1.89; *p* < 0.001) subgroups compared with the control group. The low-output subgroup showed a trend toward higher CA-AKI compared with control (HR: 1.22, 95% CI: 0.99–1.49; *p* = 0.059; Figure 3). In the patients presenting with a combination of congestion and low output, the relative frequency of AKI was similar to the congested-only subgroup (HR: 1.01; 95% CI: 0.77–1.32; *p* = 0.940).

### 3.3 Association of CA-AKI with CI, RAP, MEHRAN score, contrast agent volume, and emergency setting

The mean MEHRAN score of this cohort was 6.6 (±4.0). In univariate analysis, higher RAP was associated with increased risk of CA-AKI (HR per increase of 5 mmHg 1.14; 95% CI: 1.07–1.22; *p* < 0.001), as was a higher MEHRAN score (HR: 1.14; 95% CI: 1.12–1.16; *p* < 0.001). By contrast, CI showed no association with CA-AKI (HR: 0.95; 95% CI: 0.93–1.07; *p* = 0.358). The average volume of contrast agent was 89.9 mL. Higher contrast agent volume (HR per 10 mL increase: 1.03; 95% CI: 1.02–1.04; *p* < 0.001) and emergency setting (HR: 1.51; 95% CI: 1.26–1.81; *p* < 0.001) were each associated with higher CA-AKI rates. Rates of CA-AKI for different volumes of contrast agent are illustrated in Supplementary Figure S4. In multivariate analysis, RAP, MEHRAN score, contrast agent volume, and emergency setting were independently associated with CA-AKI (HR per increase of 5 mmHg RAP: 1.07; 95% CI: 1.01–1.15; *p* = 0.034). There was no association between CA-AKI and cardiac index (HR: 1.00; 95% CI: 0.91–1.11; *p* = 0.936). The univariate and multivariate models are illustrated in Figure 4. Sensitivity analysis using the lowest creatinine value within 7 days prior to RHC as baseline yielded comparable results (Supplementary Figure S3).

**Figure 6:**
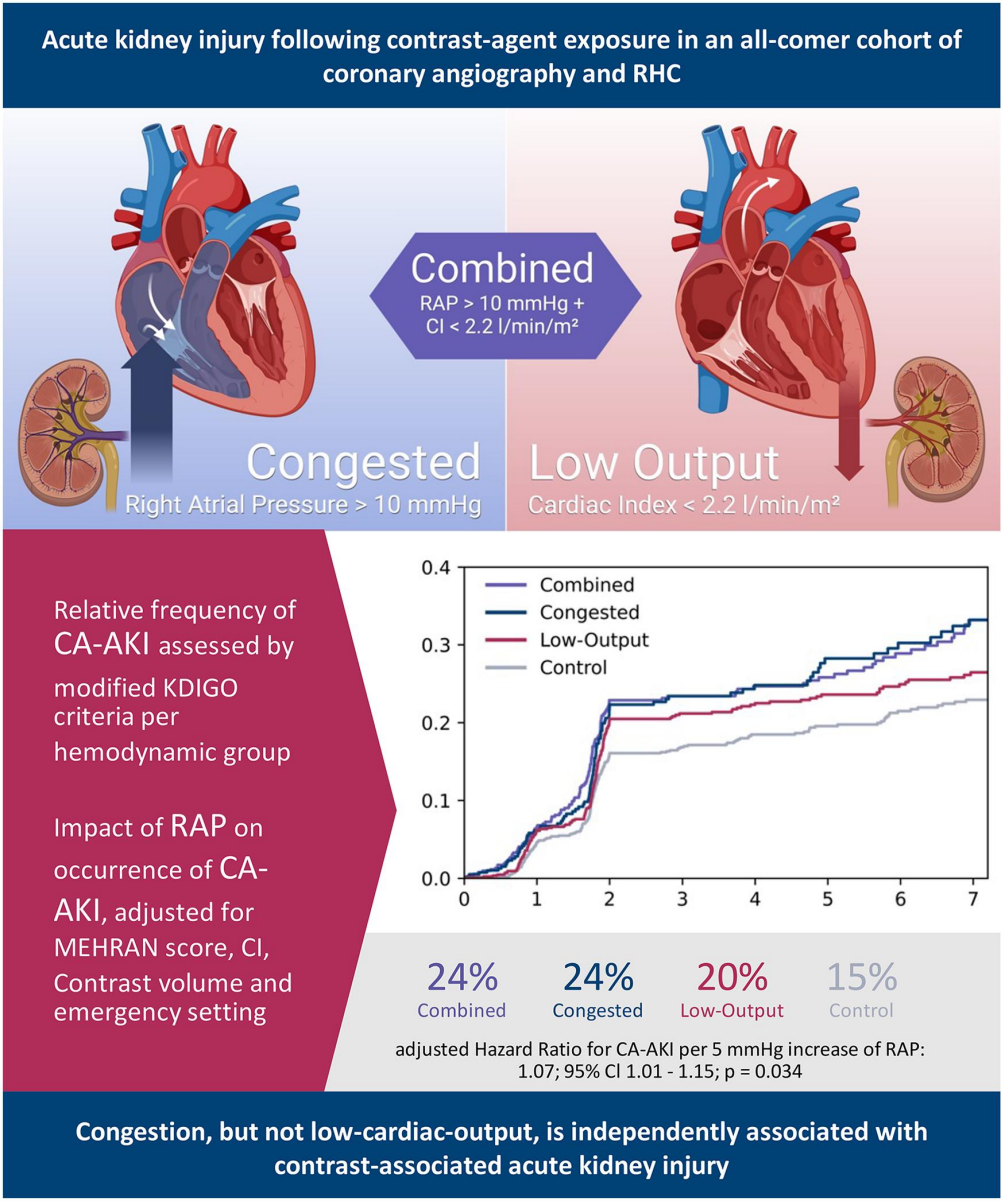
Central illustration. Contrast-associated acute kidney injury (CA-AKI) is associated with impaired clinical outcomes. We hypothesized that congestion is a stronger predictor of CA-AKI than low output in cardiovascular diseases. We divided the cohort of cases of coronary angiography and right heart catheterization into four hemodynamic subgroups (combined, congested, low output, and control). We found that CA-AKI was more frequent in patients with congestion as compared with low output. In multivariate analysis that included the MEHRAN score, only congestion, and not low output, was independently associated with CA-AKI. CA-AKI, contrast-associated acute kidney injury; CI, cardiac index; CKD, chronic kidney disease; eGFR, estimated glomerular filtration rate; KDIGO, kidney disease improving global outcomes; RAP, right atrial pressure; RHC, right heart catheterization.

 As RAP was the primary hemodynamic predictor of CA-AKI, Figure 5 illustrates the CA-AKI rate stratified by congestion status (RAP > 10 mmHg) alongside a restricted cubic spline analysis of the continuous relationship between RAP and CA-AKI risk, with hazard ratios adjusted for cardiac index and MEHRAN score. The principal findings and results of this study are presented in the central illustration in Figure 6.

### 3.4 Relationship between hemodynamics and eGFR

Independent of or simultaneous coronary angiography or contrast agent exposure, we demonstrated a curvilinear association between RAP and baseline eGFR, as well as between CI and baseline eGFR, in the cohort undergoing RHC. The maximum baseline eGFR was found within the normal ranges of RAP, between 3 and 7 mmHg, and CI, between 2.5 and 4.2 L/min/m^2^, as shown in Supplementary Figure S2. For RAP values below the normal range, there was a steep decrease in eGFR, whereas values above the normal range were associated with a milder decrease. For CI, both an increase above and a decrease below the normal range were similarly associated with a decrease in eGFR.

## 4 Discussion

The salient findings of the present study are as follows:

- Venous congestion and low cardiac output are both associated with baseline kidney function, which in turn is a factor in the occurrence of CA-AKI.
- Compared to controls, CA-AKI was more frequent in patients with venous congestion (irrespective of concurrent low cardiac output), while low cardiac output in the absence of congestion conferred a smaller trend for increase in risk.
- Venous congestion, defined as RAP >10 mmHg, but not CI, is independently associated with an increased rate of CA-AKI after adjustment for MEHRAN score, contrast agent volume, and emergency setting of coronary angiography.

These findings highlight the impact of venous congestion on renal function and occurrence of CA-AKI.

In the pathophysiology of cardio-renal syndrome, the main hemodynamic effectors are renal venous congestion and reduced organ perfusion due to low cardiac output, often referred to as forward and backward failure, respectively (18, 21). In acutely decompensated heart failure, both mechanisms simultaneously impair renal function and worsen outcomes (18). While forward failure can be partially compensated by the kidney, backward failure with fluid retention is further exacerbated by the deterioration of kidney function (18, 22). Animal studies have demonstrated the negative impact of increased renal venous pressure, primarily linked to reduced cardiac output (23, 24). The hemodynamic effects of cardiovascular disease on kidney function and development of AKI have been studied in patients with advanced heart failure, particularly emphasizing on the role of congestion (14–16, 21, 25, 26). For example, in a recent retrospective single-center cohort study of 312 children with CVD (between 2008 and 2018), evaluated for heart transplantation and congenital heart disease, an increased central venous pressure (CVP) >7 mmHg was associated with lower eGFR and decrease in renal function over 180 days after RHC. In line with our findings, CI showed no association with decrease in renal function (16). Similarly, Damman et al. conducted a large retrospective analysis investigating 2,557 cases of right heart catheterization and reported an association between CVP, CI, and eGFR, which our study reproduces (15). However, their study was limited by hemodynamic values largely within the normal range, the lack of additional variables impacting renal function, and absence of repeated creatinine measurements to apply KDIGO criteria for AKI. As the association between CVP and renal function was observed at a single point of time, the bidirectional effect of reduced fluid excretion or prior occurrence of AKI could not be accounted for. Aside from the described curvilinear relationships between hemodynamics and eGFR, which emphasize the role of congestion and low output, there is no data on the occurrence of AKI as short- and long-term prognosis-relevant renal outcomes associated with the hemodynamic state remain scarce (15). In acute right ventricular myocardial infarction, a small prospective single-center study identified right atrial pressure (RAP) > 10 mmHg as a cutoff value associated with worsening renal function (27). Contrarily, a study by Dupont et al. suggested that neither CI nor RAP affected renal function, with systemic blood pressure emerging as the central hemodynamic parameter affecting renal perfusion and function (26). Overall, the decrease in renal function and AKI is multifactorial, only partially associated with hemodynamic factors and contrast agent volume. Beyond systemic venous congestion, local renal compartment mechanisms may impair renal perfusion and tubular function independently. Increased renal interstitial pressure, arising from parenchymal edema and impaired lymphatic drainage, can compress peritubular capillaries and reduce effective filtration pressure in a manner analogous to renal compartment syndrome (28, 29).The accumulation of perirenal and renal sinus fat, increasingly prevalent in obese and cardiometabolic patients, can further compress the renal capsule and intrarenal vasculature. This elevates intrarenal pressure and worsens medullary ischemia independently of increased RAP (30). In addition, elevated intra-abdominal pressure, frequently accompanying gut congestion and ascites in decompensated heart failure or severe tricuspid regurgitation, exerts direct compressive forces on renal vasculature and the urinary collecting system, thereby impairing renal arterial inflow and venous outflow (31). Importantly, these mechanisms can coexist, interact, or mediate the effects of elevated RAP, with their relative contributions likely varying across patients.

A unique feature of our study cohort is the availability of repeated laboratory measurements and additional echocardiographic validation across a broad spectrum of CVD. A standardized, time-dependent, and prognosis-relevant outcome like AKI can be helpful in understanding the impact of hemodynamics on renal function. We defined AKI according to modified KDIGO criteria, precisely noting its onset following contrast agent exposure during coronary angiography, a widely used procedure in the assessment of CVD, known for its potential harm to renal outcomes in both the short and long term. In our cohort, we evaluated the impact of hemodynamic state on the occurrence of CA-AKI, particularly focusing on short-term changes in renal function in common scenarios of renal stress. The mean MEHRAN score of 5.8 suggests an occurrence of contrast-induced nephropathy in approximately 14% of cases in our cohort, but an AKI was observed in 19% of the cases, although the average contrast agent volume was below the threshold of 100 mL used in the MEHRAN score. To address this limitation, contrast agent volume and procedural setting were integrated into the adjusted multivariate model, as AKI more frequently occurs in cardiogenic shock, particularly due to acute coronary syndrome. We demonstrated that venous congestion, as measured by elevated RAP above 10 mmHg, was independently associated with CA-AKI, regardless of cardiac output and other known adverse factors encompassed by the MEHRAN score, emergency setting, and contrast media volume. Based on the volume of contrast agent used, the patient cohort was predominantly in groups classified at low risk (<100 mL). Furthermore, the rate of contrast volume to renal function—described in some studies as the ratio of contrast agent volume to creatinine clearance (Volume/CrCl or Volume/eGFR)—was below high-risk thresholds across all hemodynamic categories (contrast agent volume/eGFR overall 1.7, maximum in combined 1.9, minimum in control 1.5) (32, 33). Nevertheless, elevated RAP remained an independent risk factor, even in the presence of other comorbidities, such as diabetes, anemia, systolic blood pressure, age, emergency setting, and (overall low) contrast agent volume, further narrowing the safety margin for contrast agent exposure. Following AKI, the progression of CKD is a major driver of adverse outcomes not only in advanced heart failure but also in other CVDs such as valvular heart diseases, which was represented in this study. Addressing renal congestion represents one potentially modifiable component in preventing AKI and attenuating CKD progression. However, given the multifactorial nature of renal dysfunction in this setting, optimal outcomes will likely require a broader strategy that also targets underlying comorbidities, hemodynamic instability, nephrotoxic exposures, and other contributing mechanisms.

### 4.1 Limitations

This retrospective analysis of a quality assurance database has several limitations. Due to the design of the study, some cases were excluded due to missing data. The study was conducted in a single-center setting, which may limit the generalization of the findings. However, this enables interindividual comparability of the measured information, such as the MEHRAN score, as pre- and postprocedural care, medical treatment, and hydration protocols were standardized by center-specific operating procedures. For patients with eGFR <60 mL/min (CKD G3a/b), standardized preventive measures included hydration with 1 mL/kg body weight of normal saline 6–12 h prior to contrast agent exposure. For CKD G4 or G5 patients, more tailored fluid and diuretic management strategies were applied, with the goal of achieving urine output of 300 mL/h to ensure elimination of the contrast agent. The volume of contrast medium was kept to a minimum and biplane fluoroscopy was used. Nevertheless, volume administration may have contributed, in some cases, to an association between RAP and CA-AKI in patients with stage III CKD, who are therefore at risk for CA-AKI. As only database records were used, some potentially relevant information was missing, including prior contrast agent exposure from other sources (e.g., from computer tomography scans), current medications (especially diuretics), periprocedural volume administration, albuminuria, and long-term outcomes such as hospitalization for worsening heart failure. Comorbidity information regarding arterial hypertension and prevalence of diabetes had to be inferred from laboratory and hemodynamic measurements due to the absence of clinical information. This approach may in fact benefit the model, as only poorly controlled comorbidities are likely to meaningfully contribute to CKD progression and CA-AKI risk. To approximately quantify true baseline kidney function, we used the highest documented eGFR based on serum creatinine within 90 days (7 days in sensitivity analysis) prior to coronary angiography with RHC. Further information on albuminuria and serum cystatin C was missing in most cases (90%). The 90-day window was chosen to align with established diagnostic criteria for CKD and to minimize the risk of misclassification due to transiently reduced eGFR values in patients who underwent coronary angiography with RHC during an episode of acute kidney injury. To partially account for this potential bias, clinical presentation (elective vs. emergency setting) was included as a covariate. While some cases of clinically irrelevant decrease in renal function may have been missed due to early discharge of patients, these were patients at low risk of CA-AKI; cases of patients readmitted due to worsening of renal function, CA-AKI, or other complications were included in our study.

### 4.2 Clinical perspective

Volume and preload management may play an important role in determining renal outcomes, though the magnitude and mechanisms of this relationship remain incompletely understood. Decongestion of the renal venous system has been proposed as a critical therapeutic target for preserving renal function in patients with cardiovascular disease, independent of cardiac output status. This may be particularly relevant in patients with heart failure with preserved ejection fraction, in whom venous congestion is thought to contribute more prominently than low cardiac output to renal impairment—a phenomenon increasingly recognized in the literature but still underdiagnosed in clinical practice (34, 35). The association between congestion and worsening renal function was demonstrated in states of renal stress such as contrast agent exposure, which is often unavoidable in the setting of decompensation due to acute ischemic, valvular, and other cardiac disease. When pharmacologic treatment or renal replacement therapies reach their limits, the evaluation and development of devices for mechanic decongestion of the renal veins could open new avenues for preventing adverse short- and long-term outcomes. However, there are still gaps in evidence regarding the influence of congestion, which could guide therapeutic strategies aimed at preventing (CA-)AKI and mitigating progression of CKD, CVD, and their clinical sequelae (36, 37).

## 5 Conclusion

Congestion, as measured by elevated RAP, is independently associated with acute kidney injury following contrast agent exposure across a broad spectrum of cardiovascular diseases. This association was independent of the presence or absence of low cardiac output and the well-established MEHRAN score, as well as relevant comorbidities, contrast agent volume, and clinical presentation. Therefore, (a) RAP should be considered in predicting the risk of CA-AKI and (b) RAP could represent a promising therapeutic target for prevention of CA-AKI.

## Data Availability

The raw data supporting the conclusions of this article will be made available by the authors, without undue reservation.
