# Supplementary material for: Congestion, but not low cardiac output, is independently associated with acute kidney injury following contrast agent exposure
Source: Front Cardiovasc Med. 2026 Jun 15;13:1797135. doi: 10.3389/fcvm.2026.1797135 (PMC13311108; doi:10.3389/fcvm.2026.1797135)
Supplement: Supplementary file 1 [file Supplementaryfile1.docx]

Supplementary Material

# Supplementary Figures and Tables

## Supplementary tables

**Supplementary table 1:** Hemodynamic measurements and echocardiography

| **Pulmonary circulation** | | Missing values, n (%) |
| --- | --- | --- |
| RA Mean (mmHg), mean (SD) | 8.2 (5.7) | 0 |
| RA V (mmHg), mean (SD) | 12.1 (7.0) | 0 |
| RA A (mmHg), mean (SD) | 11.1 (6.8) | 0 |
| RV sys (mmHg), mean (SD) | 45.2 (15.8) | 7 (0.2) |
| RV ED (mmHg), mean (SD) | 9.1 (6.2) | 198 (6) |
| PA sys (mmHg), mean (SD) | 44.3 (16.8) | 50 (2) |
| PA mean (mmHg), mean (SD) | 28.7 (11.4) | 50 (2) |
| PA dia (mmHg), mean (SD) | 17.5 (8.9) | 50 (2) |
| PVR (dyn*s*cm^-5^), mean (SD) | 248.9 (217.5) | 574 (18) |

| **Systemic circulation and angiography** | | Missing values, n (%) |
| --- | --- | --- |
| LV sys (mmHg), mean (SD) | 153.6 (38.9) | 491 (16) |
| LV ED (mmHg), mean (SD) | 19.5 (10.1) | 503 (16) |
| AoA sys (mmHg), mean (SD) | 132.5 (31.1) | 94 (3) |
| AoA mean (mmHg), mean (SD) | 93.6 (19.2) | 94 (3) |
| AoA dia (mmHg), mean (SD) | 67.4 (16.0) | 94 (3) |
| SVR (dyn*s*cm^-5^), mean (SD) | 1637.1 (624.0) | 51 (2) |
| Cardiac Index (l/min/m^2^), mean (SD) | 2.4 (0.8) | 0 |
| Contrast agent volume (ml), mean (SD) | 89.9 (60.8) | 0 |

| **Echocardiography** | | Missing values, n (%) |
| --- | --- | --- |
| Ejection fraction (%), mean (SD) | 52.0 (14.5) | 678 (22) |
| EDV (ml), mean (SD) | 113.5 (58.2) | 627 (20) |
| ESV (ml), mean (SD) | 59.4 (48.5) | 631 (20) |
| SV (ml), mean (SD) | 67.8 (24.8) | 1699 (54) |
| LVEDD (mm), mean (SD) | 50.8 (10.1) | 593 (19) |
| LVESD (mm), mean (SD) | 38.0 (11.4) | 1471 (47) |
| IVSD (mm), mean (SD) | 12.1 (3.2) | 592 (19) |
| TAPSE (mm), mean (SD) | 19.5 (4.9) | 896 (29) |
| TRVmax (m/s), mean (SD) | 2.8 (0.7) | 2088 (67) |

Invasive hemodynamic measurements, contrast agent volume, and selected echocardiography measurements of cohort for primary exposure (N = 3126). Values are mean with standard deviation or number with relative value.

*AoA: Aorta ascendens. dia: diastolic. ED: End diastolic. EDV: End diastolic volume. ESV: End systolic volume. IVDS: Inter ventricular septum diastolic diameter. LV: Left ventricle. LVEDD: Left ventricular end diastolic diameter. LVESD: Left ventricular end systolic diameter. PA: Pulmonary atery. PVR: Pulmonary vascular resistance. RA: Right Atrium. SV: Stroke volume. SVR: Systemic vascular resistance. sys: systolic. TAPSE: Tricuspid annular plane systolic excursion. TRVmax: Tricuspid regurgitation maximum velocity.*

**Supplementary table 2:** Characteristics of full screening cohort

| Demographics | | Missing values, n (%) |
| --- | --- | --- |
| Age (years), mean (SD) | 70.1 (14.2) | 0 |
| Gender female, n (%) | 2312 (39.3) | 0 |
| BMI (kg/m^2^), mean (SD) | 27.0 (5.3) | 18 (0.3) |
| Arterial Hypertension, n (%) | 2422 (41.1) | 0 |
| Hyperlipidemia, n (%) | 1911 (32.4) | 0 |
| Diabetes, n (%) | 810 (13.8) | 0 |
| Anemia, n (%) | 1943 (33.0) | 0 |
| CKD |  | 182 (3) |
| G1, n (%) | 853 (14.5) |  |
| G2, n (%) | 2313 (39.3) |  |
| G3a, n (%) | 1138 (19.3) |  |
| G3b, n (%) | 803 (13.6) |  |
| G4, n (%) | 390 (6.6) |  |
| G5, n (%) | 209 (3.5) |  |
|  |  |  |
| Diagnose and indication for coronary angiography and RHC | | Missing values, n (%) |
| Coronary angiography, n (%)  CAD, n (%)  Acute coronary syndrome, n (%)  Out hospital cardiac arrest, n (%) | 4966 (84.3 %)  3192 (54.2 %)  740 (12.6 %)  70 (1.2 %) | 0 |
| Valvular heart disease, n (%)  Aortic stenosis, n (%)  Aortic regurgitation, n (%)  Mitral regurgitation, n (%)  Tricuspid regurgitation, n (%)  Pulmonary stenosis, n (%) | 3092 (52.2 %)  1890 (32.1 %)  127 (2.2 %)  1174 (19.9 %)  251 (4.3 %)  23 (0.4 %) | 0 |
| Heat failure, n (%)  HFrEF, n (%)  HFmrEF, n (%)  HFpEF, n (%)  DCM, n (%)  H(O)CM, n (%)  Peri-/Moycarditis, n (%)  Heart transplant, n (%)  Cardiogenic shock on inotropic, n (%)  Biopsy, n (%) | 2324 (39.5 %)  949 (16.1 %)  210 (3.6 %)  203 (3.4 %)  277 (4.7 %)  73 (1.2 %)  73 (1.2 %)  747 (12.7 %)  36 (0.6 %)  1090 (18.5 %) | 0 |
| Others, n (%)  Shunt diagnostic, n (%)  Pulmonary arterial hypertension, n (%) | 254 (4.3 %)  162 (2.8 %)  92 (1.6 %) | 0 |
|  |  |  |
| Laboratory values and scores |  | Missing values, n (%) |
| Creatinine (mg/dl), mean (SD) | 1.26 (1.0) | 191 (3) |
| eGFR (ml/min), mean (SD) | 63.0 (25.2) | 194 (3) |
| Urea (mg/dl), mean (SD) | 49.8 (29.3) | 196 (3) |
| Cystatin C (mg/l), mean (SD) | 1.9 (1.1) | 4435 (75) |
| NT-pro-BNP (pg/ml), mean (SD) | 3506 (6604) | 1342 (23) |
| HbA1c (%), mean (SD) | 6.0 (1.1) | 2366 (40) |
| Hemoglobin (g/dl), mean (SD) | 12.8 (2.1) | 180 (3) |
| Hematocrit (%), mean (SD) | 39.6 (6.0) | 180 (3) |
| MEHRAN score, mean (SD) | 6.7 (4.0) | 2762 (47) |

Characteristics of full screening cohort (N = 5888) including demographics, diagnose / indication for coronary angiography and right heart catheterization with selected diagnosis and selected laboratory values at baseline. Values are mean with standard deviation or number with relative value.

*BMI: Body mass index. CAD: Coronary artery disease. CKD: Chronic kidney disease. DCM: Dilated cardiomyopathy. HFmreF Heart failure with mid-range ejection fraction. HFpEF: Heart failure with preserved ejection fraction. HFrEF: Heart failure with reduced ejection fraction. H(O)CM: Hypertrophic (obstructive) cardiomyopathy. PAH: Pulmonary arterial hypertension.*

**Supplementary table 3:** Invasive hemodynamic measurements of full screening cohort

| Pulmonary circulation | | Missing values, n (%) |
| --- | --- | --- |
| RV sys (mmHg), mean (SD) | 42.4 (15.2) | 75 (1) |
| RV ED (mmHg), mean (SD) | 8.1 (5.9) | 375 (6) |
| PA sys (mmHg), mean (SD) | 41.3 (16.1) | 487 (8) |
| PA mean (mmHg), mean (SD) | 26.5 (11.0) | 487 (8) |
| PA dia (mmHg), mean (SD) | 15.9 (8.5) | 487 (8) |
| RA mean (mmHg), mean (SD) | 7.7 (5.6) | 413 (7) |
| RA A (mmHg), mean (SD) | 11.5 (6.7) | 408 (7) |
| RA V (mmHg), mean (SD) | 10.5 (6.7) | 408 (7) |
| PVR (dyn*s*cm^-5^), mean (SD) | 218.5 (193.5) | 1625 (28) |
|  |  |  |
| Systemic circulation and angiography | | Missing values, n (%) |
| LV sys (mmHg), mean (SD) | 153.5 (38.1) | 1812 (31) |
| LV ED (mmHg), mean (SD) | 19.0 (9.7) | 1833 (31) |
| AoA sys (mmHg), mean (SD) | 132.8 (30.7) | 1103 (19) |
| AoA mean (mmHg), mean (SD) | 94.4 (19.1) | 1103 (19) |
| AoA dia (mmHg), mean (SD) | 68.4 (16.0) | 1103 (19) |
| SVR (dyn*s*cm^-5^), mean (SD) | 1602.3 (612.9) | 120 1 (20) |
| Cardiac Index (l/min/m^2^), mean (SD) | 2.5 (0.7) | 400 (7) |
| Contrast volume (ml), mean (SD) | 81.6 (58.0) | 922 (16) |
|  |  |  |
| Echocardiography |  | Missing values, n (%) |
| Ejection fraction (%), mean (SD) | 52.7 (14.6) | 1875 (32) |
| EDV (ml), mean (SD) | 114.2 (58.8) | 1749 (30) |
| ESV (ml), mean (SD) | 59.1 (49.1) | 1775 (30) |
| SV (ml), mean (SD) | 68.6 (24.0) | 3446 (59) |
| LVEDD (mm), mean (SD) | 50 (10.3) | 1691 (29) |
| LVESD (mm), mean (SD) | 37 (11.8) | 3095 (53) |
| IVSD (mm), mean (SD) | 11 (3.2) | 1692 (29) |
| TAPSE (mm), mean (SD) | 19 (4.9) | 2206 (37) |
| Vmax TR (m/s), mean (SD) | 2.7 (0.7) | 4062 (69) |

Invasive hemodynamic measurements of full cohort (N = 5888), contrast volume, and selected echocardiography measurements. Values are mean with standard deviation or number with relative value.

*AoA: Aorta ascendens. dia: diastolic. ED: End diastolic. EDV: End diastolic volume. ESV: End systolic volume. IVDS: Inter ventricular septum diastolic diameter. LV: Left ventricle. LVEDD: Left ventricular end diastolic diameter. LVESD: Left ventricular end systolic diameter. PA: Pulmonary atery. PVR: Pulmonary vascular resistance. RA: Right Atrium. SV: Stroke volume. SVR: Systemic vascular resistance. sys: systolic. TAPSE: Tricuspid annular plane systolic excursion. TRVmax: Tricuspid regurgitation maximum velocity.*

## Supplementary Figures


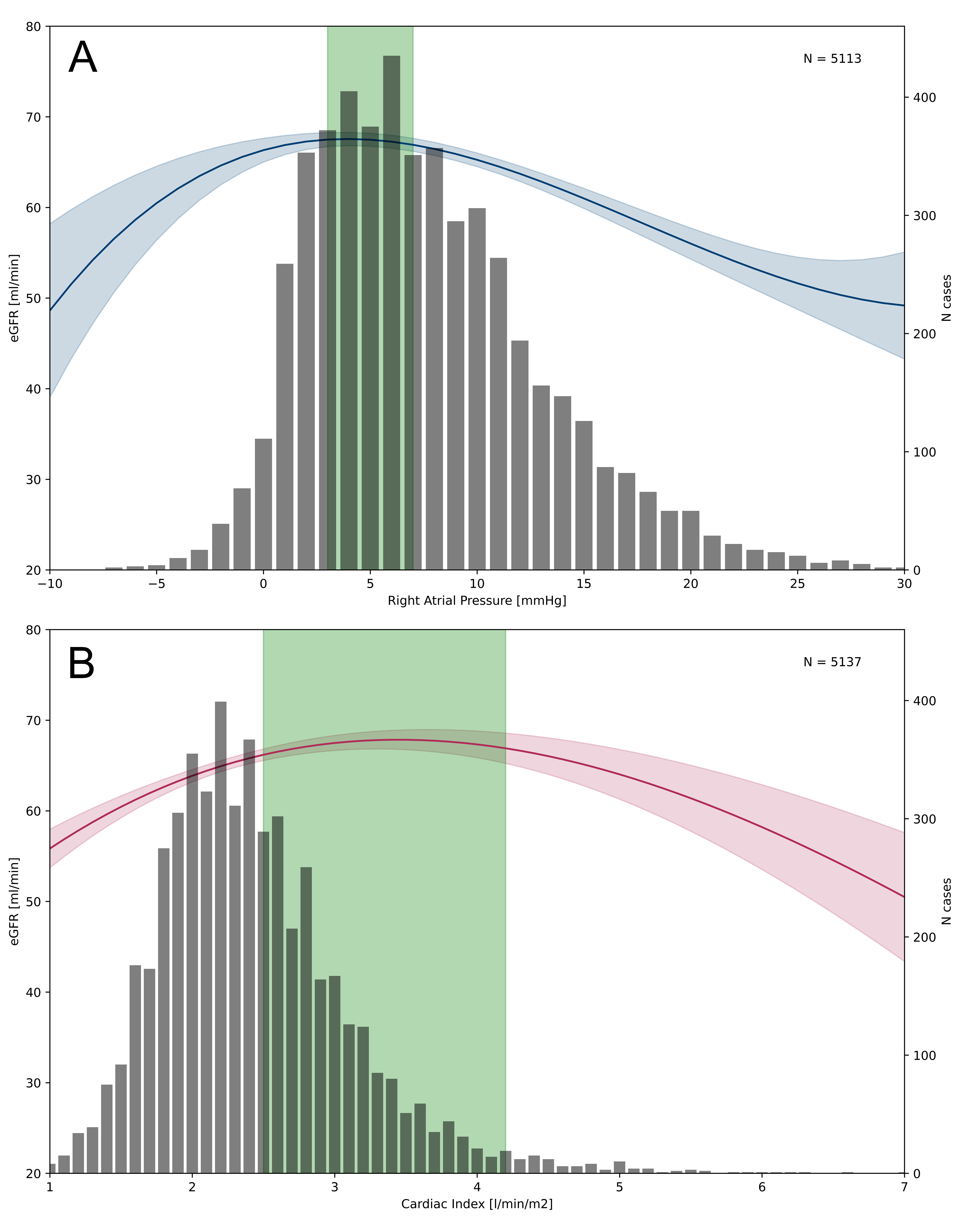
**Supplementary figure 1:** RAP, CI and eGFR. Curvilinear distribution of right atrial pressure (RAP) and eGFR (A) and cardiac index (CI) and eGFR (B) (patients with CKD V excluded). Cubic polynomial regression model using ordinary least squares method for plotting age and gender adjusted relationship between eGFR and RAP and eGFR and CI, with 95% confidence interval on the first axis. Binned number of cases for each hemodynamic measurement on the second axis. The light green area indicates normal ranges of RAP and CI. Likelihood and F-statistic suggesting non-linear relationship.

*CI: Cardiac Index. eGFR: Estimated glomerular filtration rate. RAP: Right atrial pressure.*


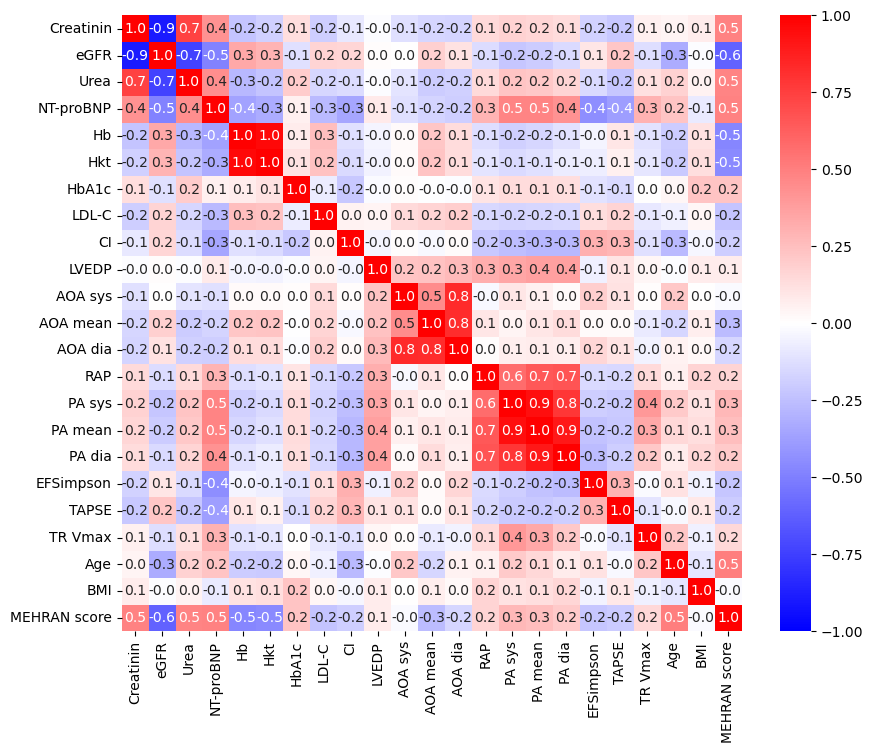


**Supplementary figure 2:** Heatmap of spearman correlations of different hemodynamic parameters, echocardiography parameters, laboratory values and demographic information in full cohort.

*AOA: Aorta ascendens. BMI: Body mass index. CI: Cardiac Index. dia: diastolic. EFSimpson: Ejection fraction measured by Simpson. eGFR: Estimated glomerular filtration rate. Hb: Hemoglobin. Hkt: Hematocrit. LDL-C: Low density lipoprotein cholesterol. LVEDP: Left ventricular end diastolic pressure. PA: Pulmonary artery. RAP: Right atrial pressure. sys: systolic. Tricuspid annular plane systolic excursion. TRVmax: Tricuspid regurgitation maximum velocity.*


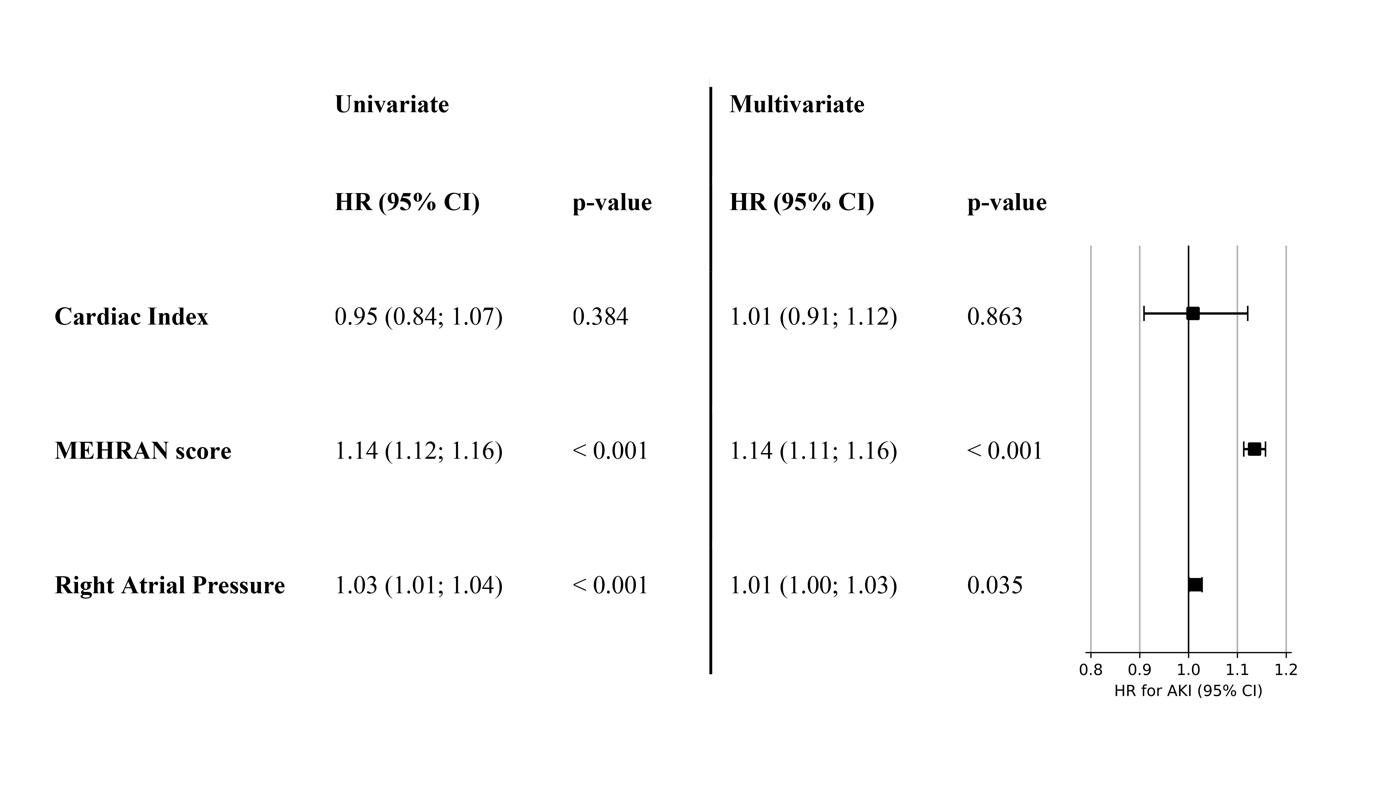


**Supplementary figure 3:** Sensitivity analysis for association of RAP and CI with CA-AKI. Univariate and multivariate cox regression analysis for outcome CA-AKI using best creatinine 7 days prior to RHC as baseline, with factors RAP, Cardiac Index and MEHRAN score (including age, systemic blood pressure, prevalence of left ventricular dysfunction, contrast media volume, diabetes mellitus, anemia, and state of chronic kidney disease).


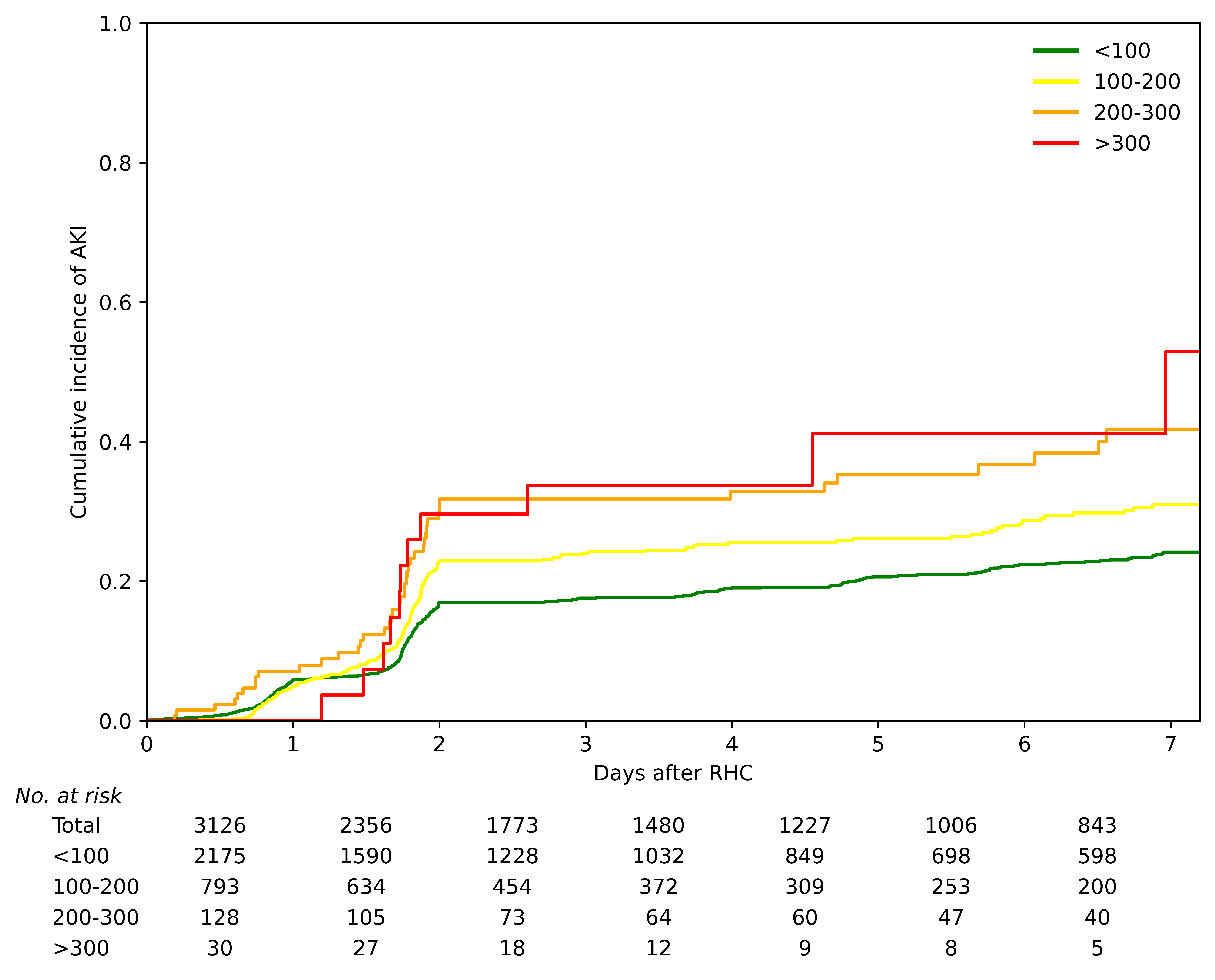


**Supplementary figure 4:** Incidence of CA-AKI stratified by contrast volume. Kaplan-Meier survival curves with cumulative incidence of contrast associated acute kidney injury (CA-AKI) within 7 days following coronary angiography with right heart catheterization in four different contrast agent volume categories. Relative frequency of CA-AKI in cases with < 100 ml of contrast agent use (n at risk at the beginning 2175) 17 %, in 100-200 ml (n at risk at the beginning 793) 23 %, 200-300 ml (n at risk at the beginning 128) 34 % and > 300 ml (n at risk at the beginning 30) 37 %. The number at risk for baseline day 0 to day 6 for the cohort (n = 3126) are listed in the table below.
